# General-Purpose Methods for Simulating Survival Data for Expected Value of Sample Information Calculations

**DOI:** 10.1177/0272989X231162069

**Published:** 2023-03-27

**Authors:** Mathyn Vervaart, Eline Aas, Karl P. Claxton, Mark Strong, Nicky J. Welton, Torbjørn Wisløff, Anna Heath

**Affiliations:** Department of Health Management and Health Economics, University of Oslo, Oslo, Norway; Department of Health Management and Health Economics, University of Oslo, Oslo, Norway; Division of Health Services, Norwegian Institute of Public Health, Oslo, Norway; Centre for Health Economics, University of York, York, UK; Department of Economics and Related Studies, University of York, York, UK; School of Health and Related Research (ScHARR), University of Sheffield, Sheffield, UK; School of Social and Community Medicine, University of Bristol, Bristol, UK; Health Services Research Unit, Akershus University Hospital, Oslo, Norway; Child Health Evaluative Sciences, The Hospital for Sick Children, Toronto, Canada; Division of Biostatistics, Dalla Lana School of Public Health, University of Toronto, Toronto, Canada; Department of Statistical Science, University College London, London, UK

**Keywords:** economic evaluation model, expected value of sample information, simulation methods, survival data, value of information

## Abstract

**Background:**

Expected value of sample information (EVSI) quantifies the expected value to a decision maker of reducing uncertainty by collecting additional data. EVSI calculations require simulating plausible data sets, typically achieved by evaluating quantile functions at random uniform numbers using standard inverse transform sampling (ITS). This is straightforward when closed-form expressions for the quantile function are available, such as for standard parametric survival models, but these are often unavailable when assuming treatment effect waning and for flexible survival models. In these circumstances, the standard ITS method could be implemented by numerically evaluating the quantile functions at each iteration in a probabilistic analysis, but this greatly increases the computational burden. Thus, our study aims to develop general-purpose methods that standardize and reduce the computational burden of the EVSI data-simulation step for survival data.

**Methods:**

We developed a discrete sampling method and an interpolated ITS method for simulating survival data from a probabilistic sample of survival probabilities over discrete time units. We compared the general-purpose and standard ITS methods using an illustrative partitioned survival model with and without adjustment for treatment effect waning.

**Results:**

The discrete sampling and interpolated ITS methods agree closely with the standard ITS method, with the added benefit of a greatly reduced computational cost in the scenario with adjustment for treatment effect waning.

**Conclusions:**

We present general-purpose methods for simulating survival data from a probabilistic sample of survival probabilities that greatly reduce the computational burden of the EVSI data-simulation step when we assume treatment effect waning or use flexible survival models. The implementation of our data-simulation methods is identical across all possible survival models and can easily be automated from standard probabilistic decision analyses.

**Highlights:**

- Expected value of sample information (EVSI) quantifies the expected value to a decision maker of reducing uncertainty through a given data collection exercise, such as a randomized clinical trial. In this article, we address the problem of computing EVSI when we assume treatment effect waning or use flexible survival models, by developing general-purpose methods that standardize and reduce the computational burden of the EVSI data-generation step for survival data.
- We developed 2 methods for simulating survival data from a probabilistic sample of survival probabilities over discrete time units, a discrete sampling method and an interpolated inverse transform sampling method, which can be combined with a recently proposed nonparametric EVSI method to accurately estimate EVSI for collecting survival data.
- Our general-purpose data-simulation methods greatly reduce the computational burden of the EVSI data-simulation step when we assume treatment effect waning or use flexible survival models. The implementation of our data-simulation methods is identical across all possible survival models and can therefore easily be automated from standard probabilistic decision analyses.

Expected value of sample information (EVSI) quantifies the expected value to a decision maker of reducing uncertainty through a given data collection exercise, such as a randomized clinical trial.^1^ Methods for computing the EVSI for collecting survival data (i.e., time-to-event data) when there is uncertainty about the choice of survival model have recently been developed by Vervaart et al.^2^ These methods require, in common with other EVSI methods, the simulation of plausible study data sets that reflect the study design proposed for collecting future data and the time-to-event distribution of individuals included in such a study.^3^ This is typically achieved by evaluating quantile functions at random uniform numbers using standard inverse transform sampling (ITS). The standard ITS method is straightforward to implement when closed-form expressions for the quantile function are available, such as for standard parametric survival models, but these are often not available when assuming treatment effect waning and for flexible survival models.

Sufficient evidence on time-to-event outcomes, such as overall survival (OS) and time to progression, is crucial for accurately determining the long-term effects of new treatments.^4^ Yet, health technology assessments often have to rely on immature survival data obtained from trials at an early stage, especially for new cancer treatments.^5^ This can partly be explained by the introduction of accelerated licensing schemes for new pharmaceuticals by regulatory bodies such as the European Medicines Agency^6,7^ and the US Food and Drug Administration.^8^ Immature survival data require a high degree of extrapolation, which led to the introduction of flexible survival models such as response-based landmark models, mixture cure models, relative survival models, and model-averaging approaches.^9,10^ Nevertheless, more complex models do not necessarily result in plausible extrapolations, and therefore, extrapolations are often supplemented with assumptions about disease progression and treatment mechanisms. For example, the National Institute for Health and Care Excellence recommends that waning of treatment effects is considered in technology appraisals,^11^ for instance, by assuming no more treatment benefit beyond a chosen time point^12^ or by assuming that the treatment effect diminishes over the long term.^13^ This is typically implemented in cost-effectiveness models by adjusting the predicted hazards, thereby altering the survival probabilities generated by parametric survival models. This poses a challenge for the EVSI data-simulation step for survival data, as closed-form expressions for the quantile function are often unavailable for custom distributions that incorporate assumptions about treatment effect waning and for flexible survival models. In these circumstances, the standard ITS method for simulating survival data could be implemented by numerically evaluating the quantile distributions at each iteration in a probabilistic analysis, but this can greatly increase the computational burden.

In this article, we address the problem of computing EVSI when we assume treatment effect waning or use flexible survival models, by developing general-purpose methods that standardize and reduce the computational burden of the EVSI data-generation step for survival data. We develop a discrete sampling method and an interpolated ITS method for simulating survival data from a probabilistic sample of survival probabilities over discrete time units. The discrete sampling method samples time cycles using the survival probabilities and sets the event times to the half-cycle times. The interpolated ITS method extends this to continuous time by initially sampling random uniform numbers between 0 and 1 and then interpolating the survival probabilities using cubic splines at the sampled numbers and recording the interpolated cycle times. We demonstrate in an illustrative case study that, when the general-purpose data-simulation methods are combined with a recently proposed nonparametric EVSI method,^2,14^ EVSI computations for survival data can be automated from standard probabilistic decision analyses, irrespective of the assumed data-generating process.

The article is structured as follows. In the second section, we first describe the standard ITS method and then introduce the general-purpose methods for simulating survival data. In the third section, we compare the standard ITS method and the general-purpose data-simulation methods based on an illustrative partitioned survival model for scenarios with and without adjustment for treatment effect waning. In the final section, we conclude with a short discussion.

## Method

### Decision Problem

In health technology assessment, cost-effectiveness models are widely used to compare alternative health technologies in terms of expected costs V and health benefits Q relative to a cost-effectiveness threshold λ .^15^ There is usually a choice between a small number of D decision options, indexed d=1,…D , such as a new treatment and standard care. The cost-effectiveness of decision option d can be expressed in terms of net health benefit, ${\mathrm{NB}}_{d}=Q_{d}-V_{d}/\lambda$ , or net monetary benefit, ${\mathrm{NB}}_{d}=Q_{d}\lambda -V_{d}$ . A cost-effectiveness model, which we denote as ${\mathrm{NB}}_{d}(\theta )$ , predicts the net benefit of decision option d , given a vector of model input parameters θ , such as probabilities, costs, and health-related quality-of-life weights. We represent our current knowledge about the cost-effectiveness model parameters in the joint probability distribution p(θ) . The joint distribution p(θ) is typically defined using a combination of statistical models fitted to individual patient data, external data sources, clinical expert opinion, and assumptions about biological plausibility, and there may also be dependency between elements of θ .

### EVSI for a New Study

The expected value of the optimal decision given current information is the value of the decision option that maximizes expected net benefit and can therefore be considered cost-effective,

(1) $$\underset{d}{\max}E_{\theta }\{{\mathrm{NB}}_{d}(\theta )\},$$

A new study would provide data X relating to θ , which could for example be a randomized controlled trial that collects OS data for a new treatment and standard care. Once the study data have been collected, we update the joint distribution of the cost-effectiveness model parameters with the new data using Bayes theorem, giving a posterior distribution p(θ|X) .

The expected value of the optimal decision made after collecting X is

(2) $$\underset{d}{\max}E_{\theta |X}\{{\mathrm{NB}}_{d}(\theta )\}.$$

The value of collecting X is derived from its potential to help a decision maker avoid recommending a treatment that reduces net benefit, as we may learn that the decision option that is considered cost-effective given current information turns out to be cost-ineffective given new information. However, as we have not yet collected X , we must take the expectation over the distribution of all possible data sets, p(X) . This requires simulating plausible data sets from the distribution of the new data, x~p(X) . To simulate x , we usually need to specify a parametric data-generating distribution, p(X|θ) , which depends on the type of data that will be generated by the proposed study and how it will be used to update the model parameters.^3^ We can simulate data sets from p(X) by first sampling from the joint distribution of the model parameters, $\theta^{*}\sim p(\theta )$ , and then sampling from the parametric data-generating distribution given the parameter sample, $x^{*}\sim p(X|\theta^{*})$ . This gives us a pair of samples $\left\{x^{*},\theta^{*}\right\}$ from the joint distribution p(X,θ) , and therefore the samples $x^{*}$ are drawn from the marginal distribution p(X) .

The expected value of the decision made with additional sample information is given by

(3) $$E_{X}[\underset{d}{\max}E_{\theta |X}\{{\mathrm{NB}}_{d}(\theta )\}].$$

The EVSI^16^ measures the expected value of reducing uncertainty about the optimal decision by collecting X , which is defined as the difference between equation (3) and equation (1),

(4) $$\mathrm{EVSI}=E_{X}[\underset{d}{\max}E_{\theta |X}\{{\mathrm{NB}}_{d}(\theta )\}]-\underset{d}{\max}E_{\theta }\{{\mathrm{NB}}_{d}(\theta )\}.$$

In the next section, we describe the EVSI estimation procedure for collecting time-to-event data.

## Computing EVSI for Time-to-Event Data

### Time-to-Event Data

Time-to-event data, such as time to disease progression and time to death, are frequently collected in the context of clinical trials. A special feature of time-to-event data is censoring, which occurs when the follow-up time is not long enough to observe the event of interest for all individuals or when individuals are lost to follow-up.^17^ A single time-to-event data set x , collected between time 0 and $t_{obs}$ , consists of i=1,…,n survival times $x_{i}$ and censoring indicators $\delta_{i}$ , $x=\{x_{1},\ldots ,x_{n},\delta_{1},\ldots ,\delta_{n}\}$ , where $\delta_{i}=1$ when $x_{i}$ is an observed event and $\delta_{i}=0$ when $x_{i}$ is a censored observation.

To predict outcomes over the long term, censored time-to-event data usually need to be extrapolated beyond the observed follow-up period using a parametric survival model.^4^ Parametric models are commonly specified using either the survivor function, S(t,θ) , or hazard function, h(t,θ) , with time denoted as t .

The survivor function S(t) defines the probability of survival up to time t , given by

(5) S(t,θ)=P(T>t)=1−F(t,θ),0<t<inf,

where F(t,θ) is the cumulative distribution function.

The hazard function h(t,θ) defines the instantaneous event rate at time t conditional on survival up to time t ,

(6) $$h(t,\theta )=\underset{\delta t\to 0}{\lim}\left\{\frac{P(t\leq T<t+\delta t\;|\;T\geq t)}{\delta t}\right\}=\frac{f(t,\theta )}{S(t,\theta )},$$

where f(t,θ) is the probability density function.

Most cost-effectiveness models are in discrete time and therefore evaluate S(t,θ) at fixed time intervals based on a set amount of time called model cycles, thereby generating a vector of survival probabilities over discrete time units for each sampled value for θ , from which the distribution of patients across the health states over time can be constructed. The expected costs, life-years, and quality-adjusted life-years of a new technology can be estimated by integrating the distribution of patients across the health states and the health state values, which reflect the costs and health-related quality-of-life weights associated with each health state.^15^

### Computing the Expected Net Benefits Given Current Information

We can compute the expected net benefits given current information in a probabilistic analysis (PA) using Monte Carlo simulation. This involves sampling k=1,…,K values, $\theta^{\left(k\right)}$ , from the distribution of the model parameters, p(θ) , and then evaluating the survivor functions, $S(t_{c},\theta^{\left(k\right)})$ , given model cycle c=0,…,C . This results in K vectors of survival probabilities, $s^{\left(k\right)}=\{s_{0}^{\left(k\right)},\ldots ,s_{C}^{\left(k\right)}\}$ , where $s_{c}^{\left(k\right)}=S(t_{c},\theta^{\left(k\right)})$ . We assume s⊂θ and compute the net benefits conditional on the survival curves and other model parameters, ${\mathrm{NB}}_{d}(\theta^{\left(k\right)})$ , for each d . We compute the expected net benefits given current information by averaging over the K net benefits for each d .

Table 1 illustrates a PA sample in which K vectors of survival probabilities $s^{\left(k\right)}$ have been generated for a single decision option d .

**Table 1:** Probabilistic Analysis Sample with K Sampled Vectors of Survival Probabilities $s^{\left(k\right)}$ for a Single Decision Option

Model Cycle	$s^{\left(1\right)}$	$s^{\left(2\right)}$	$s^{\left(3\right)}$	…	$s^{\left(K\right)}$
0	1.00	1.00	1.00	…	1.00
1	0.85	0.79	0.88	…	0.92
2	0.76	0.70	0.79	…	0.88
3	0.69	0.63	0.72	…	0.79
⋮	⋮	⋮	⋮	⋱	⋮
C	0.00	0.00	0.00	…	0.00
$\sum_{c=0}^{C}s_{c}^{\left(k\right)}$	14.49	10.82	12.11	…	18.79

## Simulating Time-to-Event Data

### Standard ITS Method

To compute the expected net benefits given new time-to-event data x , we must first sample plausible data sets from the distribution of the new data conditional on the sampled parameter values, $x^{\left(k\right)}\sim p(X|\theta^{\left(k\right)})$ . We can simulate random survival times from a parametric survival distribution using standard functions, such as the rweibull function in R.^18^ These standard functions use ITS, which is a method for generating random values from an arbitrary distribution. Let F be a continuous cumulative distribution function (CDF), with inverse CDF (i.e., quantile function) denoted by $F^{-1}$ . The intuition behind the ITS method is that if we define $T=F^{-1}(U)$ , where U~Unif[0,1] , then T is distributed according to F , that is, F(T) . Since the survivor function is defined as S(t,θ)=1−F(t,θ) , we can generate survival times by sampling values $u_{i},\;i=1,\ldots ,n$ , from a uniform distribution on the interval [0,1] and plugging these into the inverse survivor function, $S^{-1}(u_{i},\theta )$ . The inverse survivor function can be derived from the inverse CDF, $S^{-1}(1-u_{i},\theta )$ = $F^{-1}(u_{i},\theta )$ , and if U~Unif[0,1] , it follows that 1−U is also ~Unif[0,1] . We then censor the survival times at the new follow-up time $t_{new}$ .

The standard ITS scheme for simulating time-to-event data is given in Box 1.

**Box 1:** Standard Inverse Transform Sampling Scheme for Simulating Time-to-Event Data

**for k=1,…,K ** parameter samples $\theta^{\left(k\right)}$ **do** Sample a vector of values $u^{\left(k\right)}=\{u_{1}^{\left(k\right)},\ldots ,u_{n}^{\left(k\right)}\}$ , with each element drawn from Unif[0,1] Generate a dataset of n survival times $x^{\left(k\right)}=\{x_{1}^{\left(k\right)},\ldots ,x_{n}^{\left(k\right)}\}$ by plugging $u^{\left(k\right)}$ into $S^{-1}(u^{\left(k\right)},\theta^{\left(k\right)})$ Censor each element of $x^{\left(k\right)}$ at the new follow-up time $t_{new}$ **end**

Sampling from a Weibull distribution, such as by using the rweibull function in R, can result in any value between 0 and infinity. If we want to ensure that the sampled survival times do not exceed a biologically plausible time horizon $t_{h}$ , which would typically be equal to the time horizon used in the decision model, then we need to sample $u_{i}^{\left(k\right)}$ on the interval $\left[p_{1}^{\left(k\right)},1\right]$ , where $p_{1}^{\left(k\right)}$ is the survivor function evaluated at $t_{h}$ , that is, $S\left(t_{h},\theta^{\left(k\right)}\right)$ . If, however, the chance of simulating implausible survival times that exceed $t_{h}$ is substantial, the analyst should consider using a more realistic survival model, in particular models that incorporate background mortality and other external information.^9,19^ However, this evidence may not be available and is exactly the evidence that is under consideration to collect in the value-of-information analysis.

The algorithm in Box 1 could, in theory, be used for any survival model for which we can define a hazard function, h(t,θ) . The hazard could be integrated to produce a cumulative hazard function, H(t,θ) , from which we can derive the survivor function, S(t,θ)=exp[−H(t,θ)] . The survivor function could then be inverted to produce the quantile function, $S^{-1}(\cdot ,\theta )$ , and survival times could be simulated by evaluating $S^{-1}(\cdot ,\theta )$ at a random uniform sample, as described in Box 1.

Analytic solutions to the integrals and function inverses, such as implemented in the rweibull function, may not be available for flexible survival models, such as relative survival models, spline models, mixture cure models, and response-based landmark models^9,10^ and for custom distributions, including hazard functions that incorporate assumptions about treatment effect waning. In these circumstances, the integrals and function inverses could be evaluated numerically, for example, by using the integrate() and uniroot() functions in base R. The flexsurv and msm packages on CRAN also have a function qgeneric() designed to invert a generic CDF. These numerical methods, however, greatly increase the computational burden of the EVSI computations, as the integration and inversion steps need to be repeated for each of k=1,…,K simulations. In Appendix A, we provide a simple example and step-by-step implementation in R of the standard ITS method based on analytic and numerical solutions. The example illustrates that even for a simple exponential model, numerically evaluating the integrals and function inverses results in a greater than 10,000-fold increase in computational time (Table A1).

In the next section, we will introduce general-purpose methods for simulating survival data that can be standardized from standard probabilistic decision analyses and greatly reduce the computational burden of the standard ITS method when closed-form expressions for the quantile function are unavailable.

### Simulating Time-to-Event Data from a Vector of Survival Probabilities over Discrete Time Units

#### Interpolated ITS method

**Figure 1:**
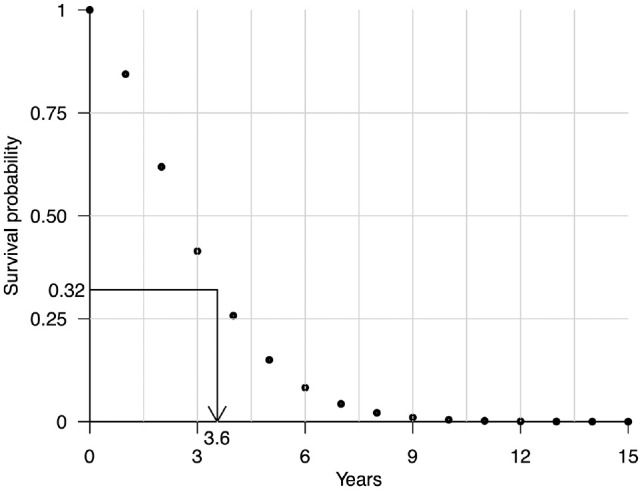
Illustration of the interpolated inverse transform sampling method for simulating time-to-event data. A survival time of 3.6 y has been simulated by first sampling a value of 0.32 from a uniform distribution between 0 and 1 and then interpolating the survival probabilities over discrete time units using monotone cubic splines at 0.32 and recording the interpolated cycle time of 3.6 y.

We can also use the ITS method to generate x from a vector of survival probabilities over discrete time units, as illustrated in Figure 1. Our PA sample consists of K vectors of survival probabilities, $s^{\left(k\right)}=\{s_{0}^{\left(k\right)},\ldots ,s_{C}^{\left(k\right)}\}$ , given model cycle c=0,…,C . We can approximate values from the quantile function by interpolating the vectors of survival probabilities, $S^{-1}(\cdot ,s^{\left(k\right)})$ . We can achieve this by fitting monotone cubic splines, a type of piecewise polynomial interpolation, to each consecutive set of survival probabilities and cycle times, $\left\{(s_{0}^{\left(k\right)},s_{1}^{\left(k\right)}),(t_{0},t_{1})\},\ldots ,\{(s_{C-1}^{\left(k\right)},s_{C}^{\left(k\right)}),(t_{C-1},t_{C})\right\}$ . We can then sample survival times from the approximate quantile function $S^{-1}(\cdot ,s^{\left(k\right)})$ using the ITS method.

The interpolated ITS sampling scheme for simulating time-to-event data from a vector of survival probabilities over discrete time units is given in Box 2.

**Box 2:** Interpolated Inverse Transform Sampling Scheme for Simulating Time-to-Event Data from a Vector of Survival Probabilities over Discrete Time Units

**for k=1,…,K ** vectors of survival probabilities $s^{\left(k\right)}=\{s_{0}^{\left(k\right)},\ldots ,s_{C}^{\left(k\right)}\}$ **do** Sample a vector of values $u^{\left(k\right)}=\{u_{1}^{\left(k\right)},\ldots ,u_{n}^{\left(k\right)}\}$ , with each element drawn from $\mathrm{Unif}[s_{C}^{\left(k\right)},1]$ Generate a dataset of n survival times $x^{\left(k\right)}=\{x_{1}^{\left(k\right)},\ldots ,x_{n}^{\left(k\right)}\}$ by interpolating $s^{\left(k\right)}$ at $u^{\left(k\right)}$ using monotone cubic splines and recording the interpolated cycle times Censor each element of $x^{\left(k\right)}$ at the new follow-up time $t_{new}$ **end**

#### Discrete sampling method

**Figure 2:**
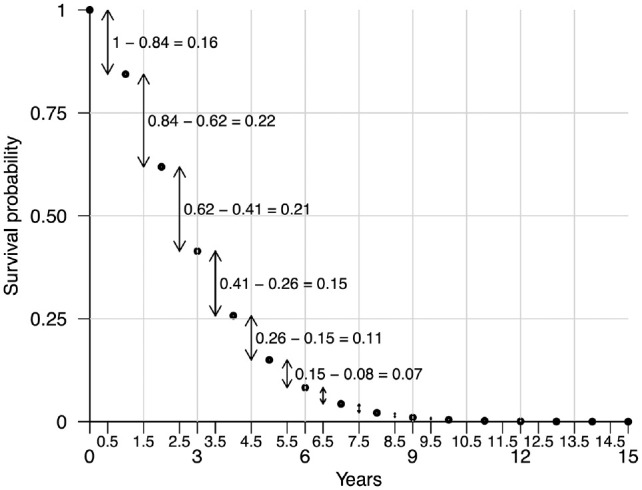
Illustration of the discrete sampling method for simulating time-to-event data. Survival times can be simulated by sampling from the half-cycle times on the *x*-axis with probability derived from the survival probabilities over discrete time units, as indicated by the arrows.

An alternative approach for simulating x from a vector of survival probabilities over discrete time units $s^{\left(k\right)}$ is by sampling discrete “bins” of cycle times with probability equal to the cumulative density of each bin, which is illustrated in Figure 2. We set the value of each bin equal to the half-cycle time $t_{c+0.5}$ , c=0,…,C−1 , that is, the midpoint in between each consecutive set of cycle times. We can estimate the cumulative density of each bin from the survival probabilities, $\left(s_{c}^{\left(k\right)}-s_{c+1}^{\left(k\right)}\right)$ . We can then generate survival times by sampling $\left\{t_{0.5},\ldots ,t_{C-0.5}\right\}$ with probability $\left\{(s_{0}^{\left(k\right)}-s_{1}^{\left(k\right)}),\ldots ,(s_{C-1}^{\left(k\right)}-s_{C}^{\left(k\right)})\right\}$ .

The discrete sampling scheme for simulating time-to-event data from a vector of survival probabilities over discrete time units is given in Box 3.

**Box 3:** Discrete sampling scheme for simulating time-to-event data from a vector of survival probabilities over discrete time units.

**for k=1,…,K ** vectors of survival probabilities $s^{\left(k\right)}=\{s_{0}^{\left(k\right)},\ldots ,s_{C}^{\left(k\right)}\}$ **do** Generate a dataset of n survival times $x^{\left(k\right)}=\{x_{1}^{\left(k\right)},\ldots ,x_{n}^{\left(k\right)}\}$ by sampling n values from the half-cycle times, $\left\{t_{0.5},\ldots ,t_{C-0.5}\right\}$ , with probability $\left\{(s_{0}^{\left(k\right)}-s_{1}^{\left(k\right)}),\ldots ,(s_{C-1}^{\left(k\right)}-s_{C}^{\left(k\right)})\right\}$ Censor each element of $x^{\left(k\right)}$ at the new follow-up time $t_{new}$ **end**

Appendix A describes a step-by-step implementation in R of the interpolated ITS and discrete sampling methods, and a comparison of their computational efficiency with the standard ITS method based on analytic and numerical solutions.

#### Computing the expected net benefits given new data

We could compute the expected net benefits given the k=1,…,K sampled data sets for each decision option, $x_{d}^{\left(k\right)}$ , using a nested Monte Carlo scheme, but this can be very computationally demanding as it requires sampling a large number of values from the posterior distribution of the model parameters conditional on each simulated data set, $p(\theta |x_{d}^{\left(k\right)})$ . A number of efficient approximation methods have therefore been developed in recent years that reduce the computational burden of the nested Monte Carlo procedure to EVSI.^20^ One of these efficient EVSI methods is a nonparametric regression-based method developed by Strong et al.^14^ that does not require a parametric distribution for the data. The regression-based method for computing EVSI relies on estimating the functional relationship between the posterior expected net benefits and the simulated data sets, thereby avoiding the need to sample from the posterior distributions $p(\theta |x_{d}^{\left(k\right)})$ , as is required for the nested Monte Carlo scheme.

In the regression-based approach, we require only the vectors of k=1,…,K prior net benefits ${\mathrm{NB}}_{d}(\theta^{\left(k\right)})$ for each decision option d that we generated in the PA, and the corresponding data sets $x_{d}^{\left(k\right)}$ that we have simulated using either of the sampling schemes above. The observed net benefits ${\mathrm{NB}}_{d}(\theta^{\left(k\right)})$ can be expressed as a sum of the conditional expectation of the net benefit given the data, $E_{\theta |x_{d}^{\left(k\right)}}\{{\mathrm{NB}}_{d}(\theta )\}$ , which we require to estimate the EVSI (equation [4]), and a mean-zero error term, $\varepsilon^{\left(k\right)}$ ,

(7) $${\mathrm{NB}}_{d}(\theta^{\left(k\right)})=E_{\theta |x_{d}^{\left(k\right)}}\{{\mathrm{NB}}_{d}(\theta )\}+\varepsilon^{\left(k\right)}.$$

Strong et al.^14^ explain that the conditional expectation $E_{\theta |x_{d}^{\left(k\right)}}\{{\mathrm{NB}}_{d}(\theta )\}$ can be thought of as an unknown function of $x_{d}^{\left(k\right)}$ . We denote this function $g(x_{d}^{\left(k\right)})$ and substitute this into equation (7), giving

(8) $${\mathrm{NB}}_{d}(\theta^{\left(k\right)})=g(x_{d}^{\left(k\right)})+\varepsilon^{\left(k\right)}.$$

We then summarize $x_{d}^{\left(k\right)}$ using a low-dimensional summary statistic for each d , $T(x_{d}^{\left(k\right)})$ ,

(9) $${\mathrm{NB}}_{d}(\theta^{\left(k\right)})=g\{T(x_{d}^{\left(k\right)})\}+\varepsilon^{\left(k\right)}.$$

A convenient choice for $T(x_{d}^{\left(k\right)})$ is the number of observed events $e_{d}^{\left(k\right)}$ and the total time at risk $y_{d}^{\left(k\right)}$ for each simulated data set $x_{d}^{\left(k\right)}$ , that is, $T(x_{d}^{\left(k\right)})=\{e_{d}^{\left(k\right)},y_{d}^{\left(k\right)}\}$ , which has been shown to give good results for various survival models.^2^

We can estimate the posterior net benefits by regressing the prior net benefits, ${\mathrm{NB}}_{d}(\theta^{\left(k\right)})$ , on the summary statistic $T(x_{d}^{\left(k\right)})$ ,

(10) $${\mathrm{NB}}_{d}(\theta^{\left(k\right)})=g\{T(x_{d}^{\left(k\right)})\}+\varepsilon ,$$

where $g_{d}$ is a function of the data $x_{d}^{\left(k\right)}$ for each d and ε is an error term with zero mean. We can achieve this by fitting a generalized additive model (GAM), which is a flexible nonparametric regression model, to each d and extracting the regression model fitted values ${\hat{g}}_{d}^{\left(k\right)}$ , which are estimates of the posterior net benefits.

The GAM-based EVSI estimate is given by

(11) $$\mathrm{EVSI}≃\frac{1}{K}\sum_{k=1}^{K}\underset{d}{\max}{\hat{g}}_{d}^{\left(k\right)}-\underset{d}{\max}\frac{1}{K}\sum_{k=1}^{K}{\hat{g}}_{d}^{\left(k\right)}.$$

#### EVSI for an ongoing study

When a trial is ongoing at the point of decision making, there could be value in reducing uncertainty by collecting additional data from the ongoing trial before making an adoption decision. This is especially common for cost-effectiveness analyses of new cancer drugs, which increasingly rely on immature data obtained from trials in an early stage.^5^

We denote the new data collected between the observed follow-up time $t_{obs}$ and future point $t_{new}$ as $\tilde{x}=\{{\tilde{x}}_{1},\ldots ,{\tilde{x}}_{\tilde{n}},{\tilde{\delta }}_{1},\ldots ,{\tilde{\delta }}_{\tilde{n}}\}$ , where $\tilde{n}$ is the number of study participants at risk at $t_{obs}$ . As we have not yet collected the new data, we need to simulate plausible new data sets conditional on the current data, $\tilde{x}\sim (\tilde{X}|X)$ . The value of extending an existing trial’s follow-up from $t_{obs}$ to future point $t_{new}$ is given by^2^

(12) $$\begin{array}{cc} \mathrm{EVSI}(\mathrm{ongoing}\;\mathrm{study}) & =E_{\tilde{X}|X}[\underset{d}{\max}E_{\theta |X,\tilde{X}}\{{\mathrm{NB}}_{d}(\theta )\}]\\ -\underset{d}{\max}E_{\theta |X}\{{\mathrm{NB}}_{d}(\theta )\}, \end{array}$$

where the first term is the expected value of a decision based on the joint posterior distribution of θ given both new data, $\tilde{X}$ , collected between $t_{obs}$ and $t_{new}$ , and current data, X , collected between time zero and $t_{obs}$ , which is computed by averaging over the posterior net benefits of the decision option that maximizes this quantity conditional on both new data and current data. The second term is the expected value of a decision based on the joint distribution of θ given current data collected up until $t_{obs}$ .

Events beyond $t_{obs}$ are conditional on survival up to $t_{obs}$ . Therefore, to simulate future survival times for $\tilde{n}$ patients at risk at $t_{obs}$ , we need to sample from a conditional distribution that is left truncated at $t_{obs}$ . For the standard ITS scheme in Box 1, this requires evaluating the survivor function at $t_{obs},\;p_{2}^{\left(k\right)}=S\left(t_{obs},\theta^{\left(k\right)}\right)$ and then sampling random uniform numbers, $u^{\left(k\right)}$ , on the interval $\left[p_{1}^{\left(k\right)},p_{2}^{\left(k\right)}\right]$ , where $p_{1}^{\left(k\right)}$ is the survivor function evaluated at the model time horizon, $S\left(t_{h},\theta^{\left(k\right)}\right)$ . For the interpolated ITS scheme in Box 2, we can find $p_{2}^{\left(k\right)}$ by interpolating $s^{\left(k\right)}$ at $t_{obs}$ and then sampling $u^{\left(k\right)}$ on the interval $\left[s_{C}^{\left(k\right)},p_{2}^{\left(k\right)}\right]$ . For the discrete sampling scheme in Box 4, we need to sample from the subset of half-cycle times that are larger than $t_{obs}$ , i.e. $\left\{t_{c+0.5},\ldots ,t_{C-0.5}\right\}$ for $t_{c+0.5}>t_{obs}$ .

#### Model-averaged EVSI

Uncertainty about the choice of survival model is often a key driver of decision uncertainty, particularly when data are immature.^21^ If we are uncertain about choosing from a set of competing survival models for extrapolating study data over the long term, $M=M_{r},\;r=1,\ldots ,R$ , then we could account for this in the EVSI computations^2^ by using model averaging.^22–24^ Before we collect new data X , our beliefs about the plausibility of each model is represented by the prior model probabilities $P(M_{r})$ . These could, for example, be derived from the Akaike’s information criterion^25^ or other measures of model fit and parsimony. In the PA, we then sample a survival model $M_{r}^{\left(k\right)}$ with probability $P(M_{r})$ before sampling $\theta_{r}^{\left(k\right)}$ from the distribution of the parameters of the sampled survival model, $p(\theta_{r},M_{r}^{\left(k\right)})$ . Since this changes only the values for $s^{\left(k\right)}$ , the discrete sampling scheme in Box 2 and interpolated ITS scheme in Box 3, as well as the GAM-based EVSI estimation following equation (11), are identical to the single-model case.

## Synthetic Case Study

### Decision Problem and Model Definition

To demonstrate the application of our methods, we developed a simple yet realistic synthetic case study based on a partitioned survival model (PSM)^26^ comparing a new treatment (*d* = 1) with standard care (*d* = 2). The PSM uses OS and progression-free survival (PFS) curves to estimate the proportion of patients in 3 health states: PFS, postprogression survival (PPS), and death, given c=0,…,360 monthly model cycles corresponding to an overall time horizon $t_{h}=30$ y. We assumed OS and PFS follow independent Weibull distributions for each d , parameterized in terms of log shape α and log scale β . The Weibull model parameters for OS are $\theta_{os1}=(\alpha_{os1},\beta_{os1})$ for the new treatment and $\theta_{os2}=(\alpha_{os2},\beta_{os2})$ for standard care, and $\theta_{pfs1}=(\alpha_{pfs1},\beta_{pfs1})$ and $\theta_{pfs2}=(\alpha_{pfs2},\beta_{pfs2})$ for PFS, respectively. We estimated the Weibull model parameters using maximum likelihood from a synthetic data set containing 100 OS times and 100 PFS times for each trial arm with a maximum follow-up of 24 mo. Further details about the synthetic case study data set are given in Appendix B. The other model parameters are utility for PFS ( $U_{pfs}$ ), utility for postprogression ( $U_{\mathrm{pps}}$ ), drug costs for the new treatment ( $C_{\mathrm{drug}1}$ ) in PFS, medical costs for the new treatment ( $C_{\mathrm{med}1}$ ), medical costs for standard care ( $C_{\mathrm{med}2}$ ), annual discount rate ( r ), and monetary value of 1 quality-adjusted life-year, ( λ ). The definitions and prior distributions for the case study model parameters are given in Table 2, and the net benefit functions are given in Appendix C.

**Table 2:** Prior Parameter Distributions for the Partitioned Survival Model Parameters

**Weibull Survival Model Parameters**		Mean , μ	**Covariance Matrix**, Σ	**Distribution**
Overall survival				
Log shape for new treatment	$\left(\begin{array}{c} \alpha_{os1}\\ \beta_{os1} \end{array}\right)$	$\left(\begin{array}{c} 0.312\\ 4.089 \end{array}\right)$	$\left(\begin{array}{cc} 0.037 & -0.036\\ -0.036 & 0.056 \end{array}\right)$	Bivariate normal (μ,Σ)
Log scale for new treatment				
Log shape for standard care	$\left(\begin{array}{c} \alpha_{os2}\\ \beta_{os2} \end{array}\right)$	$\left(\begin{array}{c} 0.361\\ 3.842 \end{array}\right)$	$\left(\begin{array}{cc} 0.029 & -0.021\\ -0.021 & 0.030 \end{array}\right)$	Bivariate normal (μ,Σ)
Log scale for standard care				
Progression-free survival				
Log shape for new treatment	$\left(\begin{array}{c} \alpha_{pfs1}\\ \beta_{pfs1} \end{array}\right)$	$\left(\begin{array}{c} 0.161\\ 3.590 \end{array}\right)$	$\left(\begin{array}{cc} 0.019 & -0.010\\ -0.010 & 0.021 \end{array}\right)$	Bivariate normal (μ,Σ)
Log scale for new treatment				
Log shape for standard care	$\left(\begin{array}{c} \alpha_{pfs2}\\ \beta_{pfs2} \end{array}\right)$	$\left(\begin{array}{c} 0.209\\ 3.294 \end{array}\right)$	$\left(\begin{array}{cc} 0.014 & 0.004\\ -0.004 & 0.013 \end{array}\right)$	Bivariate normal (μ,Σ)
Log scale for standard care				
**Utility parameters**		**Mean**, μ	**Standard error, SE**	**Distribution**
Utility for progression-free survival	$U_{pfs}$	0.80	0.04	Beta(80,20)
Utility for post-progression survival	$U_{\mathrm{pps}}$	0.50	0.05	Beta(50,50)
**Cost parameters (monthly)**		**Mean** μ	**Standard error, SE**	**Distribution**
Drug costs for new treatment	$C_{\mathrm{drug}1}$	1, 200	—	Constant
Medical costs for new treatment	$C_{\mathrm{med}1}$	500	250	Gamma(4,0.008)
Medical costs for standard care	$C_{\mathrm{med}2}$	500	250	Gamma(4,0.008)
**Other parameters**		**Mean**, μ	**Standard error, SE**	**Distribution**
Annual discount rate	r	0.035	—	Constant
Monetary value of 1 quality-adjusted life-year	λ	80, 000	—	Constant

#### Treatment-stopping rule and treatment effect waning

We also considered a scenario with a 2-y treatment-stopping rule, after which the drug costs for the new treatment $C_{\mathrm{drug}1}=0$ . We assumed that, on expectation, the treatment effect on OS and PFS would wane after stopping treatment at year 2 until there was no more treatment effect by year 4. A treatment effect waning is commonly implemented in cost-effectiveness models by setting the hazards in the treatment arm and comparator arm equal at a chosen time point. This approach has a number of limitations. First, this could lead to counterintuitive results when the hazard in the comparator arm is below the hazard in the treatment arm after the treatment duration cutoff, in which case the treatment effect increases during the waning period.^12^ Second, setting the hazards in the treatment and comparator arm equal underestimates uncertainty about independent survival endpoints. Third, this approach ignores uncertainty about the start time and duration of the waning period.

To avoid these limitations, we used an alternative approach to implement treatment effect waning. For k=1,…,K , we first sampled values, $t_{w1}^{\left(k\right)}$ and $t_{w2}^{\left(k\right)}$ , for the start time and duration of the waning period from $p(t_{w1})\sim \mathrm{LogNormal}(2.83,0.69)$ and $p(t_{w2})\sim \mathrm{LogNormal}(2.83,0.69)$ , corresponding to mean times and standard deviations of 24 months for both $t_{w1}$ and $t_{w2}$ . In practical applications, $p(t_{w1})$ and $p(t_{w2})$ could be estimated from previous studies or by expert elicitation.^27^ We then computed vectors of “waning hazards” from the Weibull mean survival probabilities for the new treatment and standard care that in linear proportions increase from no waning to full waning over the time periods defined by $t_{w1}^{\left(k\right)}$ and $t_{w2}^{\left(k\right)}$ , and added these to the sampled Weibull hazards for the new treatment, $h_{1}^{\left(k\right)}$ .

The waning-adjusted hazard function for OS and PFS for the new treatment is given by

(13) $$\begin{array}{c} \mathrm{Waning}-\mathrm{adjusted}\;\mathrm{hazard}h_{1}^{*}(t)=\frac{e^{\theta_{\alpha 1}}}{e^{\theta_{\beta 1}}}{\left(\frac{t}{e^{\theta_{\beta 1}}}\right)}^{e^{\theta_{\alpha 1}}-1}\\ +F_{Unif}\left(\frac{t-t_{w1}}{t_{w2}}\;|\;0,1\right)\max[0,\;f_{spline}(t,\;{\hat{h}}_{2}-{\hat{h}}_{1})], \end{array}$$

where the first term is the Weibull hazard function, $F_{Unif}(\;\cdot \;|\;0,1)$ is a uniform cumulative distribution function on the interval [0,1] evaluated at $(t-t_{w1})/t_{w2}$ , and $f_{spline}(t,\;{\hat{h}}_{2}-{\hat{h}}_{1})$ is a spline function fitted to the difference in mean survival hazards for the new treatment and standard care, ${\hat{h}}_{1}$ and ${\hat{h}}_{2}$ , respectively, with mean survival hazards for treatment option d given by

(14) $$\mathrm{Mean}\;\mathrm{survival}\;\mathrm{hazards}\;{\hat{h}}_{d}=-\log\left(1-\frac{\left\{{\bar{s}}_{d,1},\ldots ,{\bar{s}}_{d,C}\right\}}{\left\{{\bar{s}}_{d,0},\ldots ,{\bar{s}}_{d,C-1}\right\}}\right),$$

where ${\bar{s}}_{d,c}$ , c=0,…,C , are mean Weibull survival probabilities over discrete time units for each d , given by

(15) $$\mathrm{Mean}\;\mathrm{survival}\;\mathrm{probability}\;{\bar{s}}_{d,c}=\frac{1}{K}\sum_{k=1}^{K}e^{-{\left(t_{c}/e^{\beta_{d}^{\left(k\right)}}\right)}^{e^{\alpha_{d}^{\left(k\right)}}}}.$$

All other model assumptions and net benefit functions are as above.

**Figure 3:**
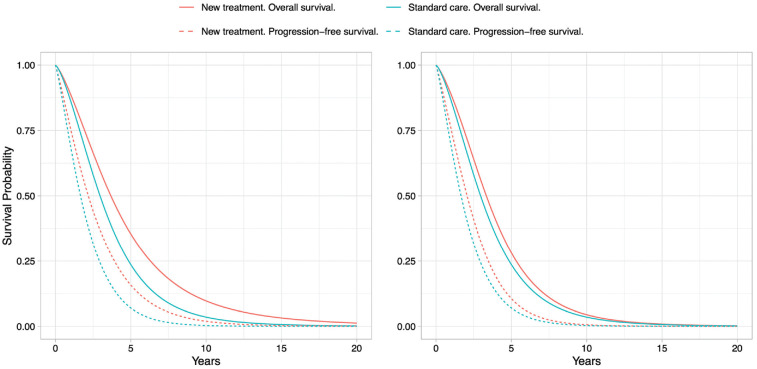
Expected Weibull survival curves for overall survival and progression-free survival for the new treatment and for standard care without adjustment (left) and with adjustment for treatment effect waning (right).

The expected Weibull survival curves for the scenarios with and without adjustment for treatment effect waning are given in Figure 3.

### Computations

We assume we want to compute the EVSI for a new study that will collect OS and PFS data for the new treatment and for standard care. We considered a sample size of n=100 study participants for each treatment arm and a study follow-up period $t_{new}=1,2,3,4,5$ and 10 y. We first performed a PA in which we sampled K=2,000 parameter values $\theta^{\left(k\right)}$ from the distribution of the model parameters p(θ) and then evaluated the PSM to obtain the net benefits for each d , ${\mathrm{NB}}_{d}(\theta^{\left(k\right)})$ .

#### Simulating OS and PFS data

In the scenario without adjustment for treatment effect waning, we simulated OS data sets, $x_{os1}^{\left(k\right)}$ for the new treatment and $x_{os2}^{\left(k\right)}$ for standard care, with n=100 times for each treatment arm using the standard ITS method, the interpolated ITS method and the discrete sampling method following the sampling schemes in Box 1, Box 2, and Box 3, respectively. In the scenario with adjustment for treatment effect waning, we implemented the standard ITS method by numerically integrating the waning hazard function and inverting the waning survivor function, as analytic solutions are unavailable. The implementation of the interpolated ITS method and the discrete sampling method is identical to the scenario without treatment effect waning. We summarized each simulated data set using the number of observed OS events, $e_{os1}^{\left(k\right)}$ and $e_{os2}^{\left(k\right)}$ , and the total time at risk for OS, $y_{os1}^{\left(k\right)}$ and $y_{os2}^{\left(k\right)}$ , that is, $T(x_{os1}^{\left(k\right)})=\{e_{os1}^{\left(k\right)},y_{os1}^{\left(k\right)}\}$ and $T(x_{os2}^{\left(k\right)})=\{e_{os2}^{\left(k\right)},y_{os2}^{\left(k\right)}\}$ .

We simulated PFS data sets for the new treatment and standard care ( $x_{pfs1}^{\left(k\right)}$ and $x_{pfs2}^{\left(k\right)}$ ) using the same sampling schemes as for OS for the scenarios with and without adjustment for treatment effect waning. To prevent double counting of OS data, we censored the simulated PFS times at the proposed follow-up time or at the time point at which, if at all, the PFS curve crosses and is set equal to the OS curve, whichever is soonest. We then computed the number of observed PFS events, $e_{pfs1}^{\left(k\right)}$ and $e_{pfs2}^{\left(k\right)}$ , and the total time at risk for PFS, $y_{pfs1}^{\left(k\right)}$ and $y_{pfs2}^{\left(k\right)}$ , for each simulated PFS data set, that is, $T(x_{pfs1}^{\left(k\right)})=\{e_{pfs1}^{\left(k\right)},y_{pfs1}^{\left(k\right)}\}$ and $T(x_{pfs2}^{\left(k\right)})=\{e_{pfs2}^{\left(k\right)},y_{pfs2}^{\left(k\right)}\}$ .

#### Computing EVSI via GAM regression

To reduce the number of regression equations^14^ and improve the stability of the EVSI computations,^28^ we used the incremental net benefit (INB), defined as $\mathrm{INB}(\theta^{\left(k\right)})={\mathrm{NB}}_{1}(\theta^{\left(k\right)})-{\mathrm{NB}}_{2}(\theta^{\left(k\right)})$ . We estimated posterior INB by fitting a single GAM model with $\mathrm{INB}(\theta^{\left(k\right)})$ as the dependent variable and separate sets of summary statistics per treatment arm, $\left\{e_{os1}^{\left(k\right)},y_{os1}^{\left(k\right)},e_{pfs1}^{\left(k\right)},y_{pfs1}^{\left(k\right)}\right\}$ and $\left\{e_{os2}^{\left(k\right)},y_{os2}^{\left(k\right)},e_{pfs2}^{\left(k\right)},y_{pfs2}^{\left(k\right)}\right\}$ , as the independent variables. We implemented the GAM regression using the R package mgcv^29^ and specified a tensor product cubic regression spline basis for the independent variables, with a maximum basis dimension of 4 to prevent the model from estimating too many coefficients. This has syntax gam (inb∼te(e_os1, y_os1, e_pfs1, y_pfs1, k=4) + te(e_os2, y_os2, e_pfs2, y_pfs2, k=4)). We extracted the GAM model fitted values, ${\hat{g}}^{\left(k\right)}$ , which are estimates of the posterior INB, and estimated EVSI using the equation given by

(16) $$\mathrm{EVSI}\approx \frac{1}{K}\sum_{k=1}^{K}\max\{0,{\hat{g}}^{\left(k\right)}\}-\max\frac{1}{K}\sum_{k=1}^{K}\{0,{\hat{g}}^{\left(k\right)}\}.$$

We computed 95% intervals for the GAM estimator by sampling 2,000 values from a multivariate normal distribution of the GAM coefficients, as described in an appendix of the article by Strong et al.^30^

## Results

**Figure 4:**
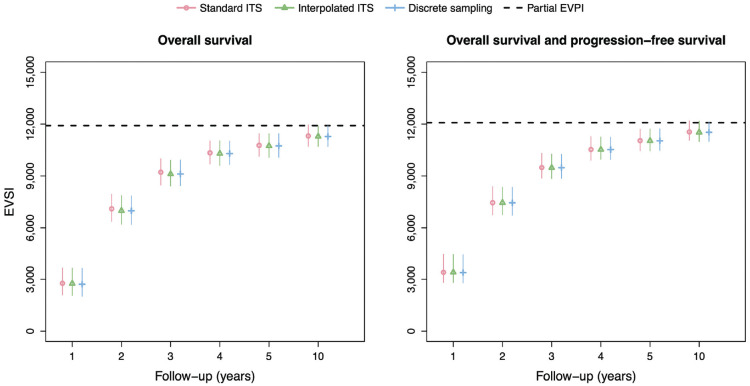
EVSI values for the synthetic case study without adjustment for treatment effect waning. Total computation times for the data-simulation procedures are 12 s (standard ITS), 32 s (interpolated ITS) and 12 s (discrete sampling). EVSI, expected value of sample information; ITS, inverse transform sampling.

Figure 4 shows the EVSI values and 95% intervals without adjustment for treatment effect waning for follow-up times of 1, 2, 3, 4, 5, and 10 y. There is excellent agreement between the standard ITS method, interpolated ITS method, and discrete sampling method both for OS only and OS and PFS. The EVSI reflects diminishing marginal returns for increasing follow-up durations, ranging from 2,711 to 11,311 for OS only and from 3,387 to 11,547 for OS and PFS, and converges toward the partial EVPI for the respective sets of model parameters. This indicates that the value of reducing uncertainty about PFS in addition to OS is relatively small. We did not compute the EVSI for PFS only, since PFS is a composite endpoint that is defined as time to progression or time to death, whichever is soonest, and therefore also requires the collection of OS data. The total computation times for the data-simulation procedures in the scenario without adjustment for treatment effect waning are 12 s, 32 s, and 12 s for the standard ITS method, interpolated ITS method, and discrete sampling method, respectively.

**Figure 5:**
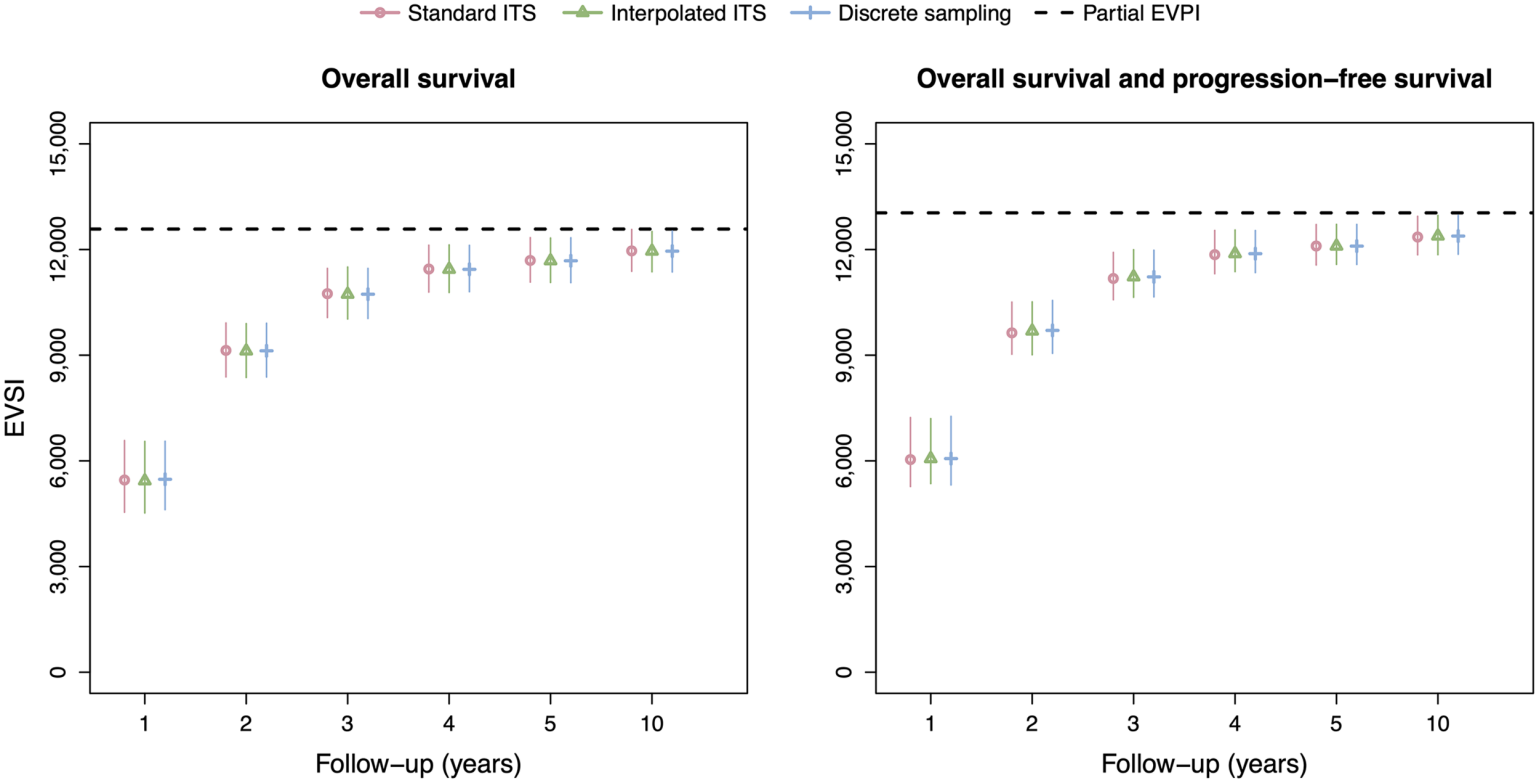
EVSI values for the synthetic case study with adjustment for treatment effect waning. Total computation times for the data-simulation procedures are 120,180 s (standard ITS), 33 s (interpolated ITS), and 12 s (discrete sampling). EVSI, expected value of sample information; ITS, inverse transform sampling.

The EVSI estimates in the scenario with adjustment for treatment effect waning (Figure 5) are greater than in the scenario without adjustment for treatment effect waning, reflecting the added value of learning about treatment effect waning. The EVSI estimates range from 5,433 to 11,961 for OS only and from 6,064 to 12,384 for OS and PFS, almost twice as high for the 1-y follow-up period compared with the scenario without adjustment for treatment effect waning. The interpolated ITS method and discrete sampling method again agree closely with the standard ITS method but at a greatly reduce computational cost. The computation times for the interpolated ITS and discrete sampling methods are, in fact, the same as in the scenario without adjustment for treatment effect waning, and approximately 3,600 and 10,000 times faster, respectively, than the standard ITS scheme that used numerical solutions for the integrals and function inverses.

## Discussion

### Strengths and Limitations

We developed an interpolated ITS method and a discrete sampling method for simulating survival data from a probabilistic sample of survival probabilities over discrete time units. Our general-purpose methods greatly reduce the computational burden of the standard ITS method when closed-form expressions for the quantile function are unavailable, such as for custom distributions that incorporate assumptions about treatment effect waning as commonly encountered in practice,^11^ and for flexible survival models, including relative survival models, spline models, mixture cure models, and response-based landmark models.^9,10^ The implementation of our methods is identical across all possible survival models and can therefore be easily standardized from standard probabilistic decision analyses.

Generally, the precision of the EVSI estimator is influenced by the number of simulated data sets and the effective sample size of the simulated data. The discrete sampling method and, to a lesser degree, the interpolated ITS method, additionally introduce an approximation error that depends on the cycle length. It is generally recommended that discrete-time health economic models use a short cycle length to reduce the discrete-time approximation error, which could be as short as 1 week for slowly progressing chronic diseases.^31^ Our synthetic case study suggests that the approximation error introduced by our general-purpose methods is very small even when using a longer cycle length of 1 mo in combination with short follow-up times and a low effective sample size of the simulated data given a rapidly progressing disease.

We structured the synthetic case study around a PSM, a type of model that is frequently used to inform reimbursement decisions for new oncology drugs.^27^ The key assumption behind a PSM is that survival endpoints, such as OS and PFS, are independent. This also implies that dependency between OS and PFS is not reflected in the EVSI data-simulation procedure when using a PSM. Joint modeling of OS and PFS could be implemented in a state transition model (STM), which uses transition probabilities to describe movements between health states over time. STMs require individual patient data to estimate all relevant transition probabilities, unlike PSMs, which can use digitized Kaplan–Meier data from published trials. OS and PFS data can be simulated jointly from a STM by first sampling a PFS time and then deciding whether the sampled PFS time is a progression or death event using a binomial experiment with probability derived from the hazards of transitioning from PFS to PPS and OS.^32^ If the sampled PFS time is a progression event, residual time until death can be simulated using the survival distribution for PPS to OS. Since transition probabilities are typically derived from survival curves fitted to time-to-event data, our data-simulation methods could also be useful in a STM framework.

If individual patient data are available with a similar study design and at least the same length of follow-up as the proposed study, study data sets could alternatively be simulated using a 2-level resampling method based on bootstrapping.^33^ In this approach, the observed data set is first resampled K times with replacement, and then n values are sampled with replacement from the k=1,...,K resampled data sets, thereby generating K new data sets with n observations each. In most situations, particularly for novel treatments, this type of data will, however, not be available.

The key notion behind EVSI is that the prior distribution of the model parameters is updated with simulated study data to estimate the joint posterior distribution given both prior information and the simulated study data. In EVSI analyses, the prior distribution is often informed by external evidence, such as digitized Kaplan–Meier data. This may, however, not match the way in which real-world analyses of study data are conducted, since these may not synthesize the collected study data and the external evidence. Analysts should therefore ensure that the way in which the study data is analyzed once it has been collected is aligned with the assumptions underpinning the EVSI analysis.

Despite its routine application in cost-effectiveness analyses, there is currently a lack of guidance on how to model treatment effect waning.^12^ In the synthetic case study, we modeled treatment effect waning by specifying probability distributions for the start and duration of the waning period, while preserving uncertainty about independent survival endpoints using a novel additive hazard approach. This had a large impact on the EVSI estimates, which highlights the importance of appropriately incorporating uncertainty about treatment effect waning in the EVSI calculations. There may be other possible approaches to model treatment effect waning, and these can easily be captured by our data-simulation methods as well.

## Conclusion

The increasing prevalence of immature survival data in decision making, particularly for new cancer treatments,^5^ has been accompanied by the introduction of increasingly complex approaches for extrapolation,^9,10^ which complicates the EVSI data-simulation step. Our general-purpose data-simulation methods greatly reduce the computational burden of the EVSI data-simulation step when custom distributions that incorporate treatment effect waning or flexible survival models are used for which closed-form expressions for the quantile function are unavailable. Our methods are straightforward to implement and can easily be automated from standard probabilistic decision analyses, such as those used in technology assessments of new pharmaceuticals.^11,34–36^ This means that our general-purpose methods can be used to simulate survival data—with a similar accuracy and computational cost—as using the correct closed-form quantile function for any survival model. Efficient EVSI calculations for survival data can help decision makers determine whether current evidence is sufficient or whether there is a need for collecting additional survival data before making an adoption decision.

## Supplemental Material

sj-docx-1-mdm-10.1177_0272989X231162069 – Supplemental material for General-Purpose Methods for Simulating Survival Data for Expected Value of Sample Information Calculations Supplemental material, sj-docx-1-mdm-10.1177_0272989X231162069 for General-Purpose Methods for Simulating Survival Data for Expected Value of Sample Information Calculations by Mathyn Vervaart, Eline Aas, Karl P. Claxton, Mark Strong, Nicky J. Welton, Torbjørn Wisløff and Anna Heath in Medical Decision Making
