# Supplementary material for: General-Purpose Methods for Simulating Survival Data for Expected Value of Sample Information Calculations
Source: Med Decis Making. 2023 Mar 27;43(5):595–609. doi: 10.1177/0272989X231162069 (PMC10336715; doi:10.1177/0272989X231162069)
Supplement: sj-docx-1-mdm-10.1177_0272989X231162069 – Supplemental material for General-Purpose Methods for Simulating Survival Data for Expected Value of Sample Information Calculations [file sj-docx-1-mdm-10.1177_0272989X231162069.pdf]

General-Purpose Methods for Simulating Survival Data for Expected Value of Sample Information Calculations. Supplementary online material.

Mathyn Vervaart[1,*], Eline Aas[1], Karl P. Claxton[2,3], Mark Strong[4], Nicky J. Welton[5], Torbjørn Wisløff[6], Anna Heath[7,8,9]

[1]Department of Health Management and Health Economics, University of Oslo, Oslo, Norway
[2]Centre for Health Economics, University of York, York, UK
[3]Department of Economics and Related Studies, University of York, York, UK
[4]School of Health and Related Research (ScHARR), University of Sheffield, Sheffield, UK
[5]School of Social and Community Medicine, University of Bristol, Bristol, UK
[6]Health Services Research Unit, Akershus University Hospital, Oslo, Norway
[7]Child Health Evaluative Sciences, The Hospital for Sick Children, Toronto, Canada
[8]Division of Biostatistics, Dalla Lana School of Public Health, University of Toronto, Toronto, Canada.
[9]Department of Statistical Science, University College London, London, UK

Appendix A - Methods for Simulating Survival Times

In this appendix we will illustrate how to simulate survival times in `R` using the 3 methods discussed in the paper: standard inverse transform sampling (standard ITS), interpolated inverse transform sampling (interpolated ITS) and discrete sampling.

We will first install (if not installed yet) and load the `flexsurv`, `tidyverse` and `knitr` packages, which we will use in the example.

```
if ("flexsurv" %in% rownames(installed.packages()) == FALSE) {
  install.packages("flexsurv") # install flexsurv if not installed yet
}
if ("tidyverse" %in% rownames(installed.packages()) == FALSE) {
  install.packages("tidyverse") # install tidyverse if not installed yet
}
if ("knitr" %in% rownames(installed.packages()) == FALSE) {
  install.packages("knitr") # install flexsurv if not installed yet
}
library(flexsurv) # load flexsurv
library(tidyverse) # load tidyverse
library(knitr) # load knitr
```

*Corresponding author: Mathyn Vervaart, Department of Health Management and Health Economics, University of Oslo, Forskningsveien 3A, Harald Schjelderups hus, 0373 Oslo, Norway (mathyn.vervaart@medisin.uio.no).

Probabilistic Analysis

We start by running a probabilistic analysis in which we sample $k = 1, \ldots, K$ values $\boldsymbol{\theta}^{(k)}$ from the prior distribution of the model parameters, $p(\boldsymbol{\theta})$, which in this example is the rate parameter of an exponential model that describes overall survival for a single treatment arm.

```
set.seed(1) # set the seed for reproducibility
K <- 1000 # number of simulations
theta <- rgamma(K, 35, 950) # prior distribution for the exponential rate
```

We then construct K vectors of survival probabilities by evaluating the exponential survivor function given the sampled values $\boldsymbol{\theta}^{(k)}$ at discrete time cycles.

```
cycles <- 0:240 # discrete time cycles
surv <- sapply(theta, function (x) {
  pexp(cycles, x, lower.tail = F) # evaluate the survivor function
})
```

The following code produces a plot of the mean survival curve and 95% bounds.

```
# compute 95% percentiles
ci <- sapply(cycles+1, function (x) {quantile(surv[x,], c(0.025, 0.975))})

# create a dataframe of the sampled survival probabilities and confidence bounds
df_surv <- data.frame(
  "Time" = cycles,
  "Survival" = rowMeans(surv),
  "Upper" = ci[2, ],
  "Lower" = ci[1, ]
)

# plot mean survival and 95% confidence bounds
ggplot(df_surv, aes(Time, Survival)) +
  geom_ribbon(aes(ymin = Lower,
                  ymax = Upper),
                  fill = "firebrick",
                  alpha = 0.2) +
  geom_line(color = "firebrick")
```

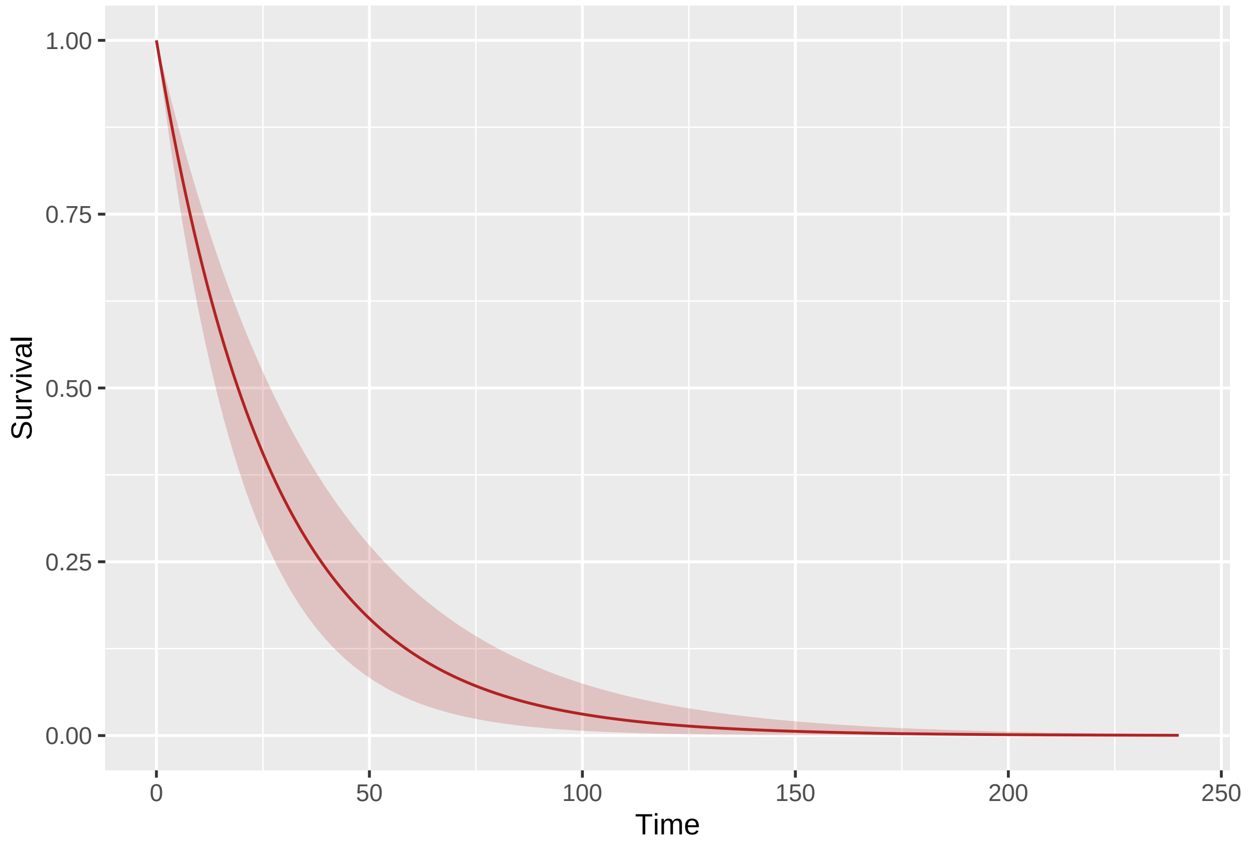

Standard Inverse Transform sampling

Simulating Survival Data for a New Study

We can simulate survival times for a new study using the standard inverse transform sampling method by evaluating the quantile function at random uniform samples between 0 and 1. We can implement this using the `rexp` function in base `R`, which requires 2 arguments: the number of observations (e.g. number of patients to be included in the new study) and a value for the exponential rate parameter.

```
n <- 100 # number of patients/observations

# simulate n survival times given theta
times_std1 <- sapply(theta, function (x) rexp(n, x) )
```

The output is a matrix of simulated survival times, where rows index the n simulated survival times, and columns index the K simulations. The first 10 simulated survival times for the first 5 simulations are given by:

```
times_std1[1:10, 1:5]
##             [,1]      [,2]      [,3]      [,4]       [,5]
##  [1,] 22.5218924  3.943437  4.373529  8.961688  42.061293
##  [2,] 24.0482783  5.001096  8.227731 59.718875   9.319299
##  [3,]  0.7739475 32.194231  2.212844 16.903244  49.725231
##  [4,]  4.1427170 40.615923  5.390183 90.057154 142.905871
##  [5,] 39.9443093 52.405560 19.684164 62.166050   6.600515
```

```
## [6,] 29.8874133 17.284868 18.631781  5.463462  26.606568
## [7,] 22.5574594 20.743536  4.572290  5.475647 107.326405
## [8,] 19.8110560 35.331819 38.729165 12.121705  51.730617
## [9,]  6.4400761  4.422201 11.617468 42.017328  52.588304
## [10,] 10.5242127  1.511484 21.624664  9.374594   2.223138
```

Simulating Survival Data for an Ongoing Study

The `rexp` function simulates survival times that can take any value between 0 and infinity. If we want to simulate survival times for an ongoing study, we need to sample survival times that are greater than the observed follow-up time t_{obs}. We can do this by sampling from a conditional quantile function that is left-truncated at t_{obs}. We can implement this in `R` using the following code (we will record the computation time using the `Sys.time()` function):

```
# generate hypothetical observed follow-up times for the n patients
t_obs <- runif(n, 8, 12)

start_t <- Sys.time() # start the clock

# evaluate the survivor function at t_obs given theta
p_2 <- sapply(theta, function (x) pexp(t_obs, x, lower.tail = F))

# sample random numbers on the interval [0, p_2]
r_num <- sapply(1:K, function (x) runif(n, 0, p_2[,x]))

# evaluate the quantile function given theta at the random uniform values
times_std2 <- sapply(1:K, function (x) qexp(r_num[,x], theta[x], lower.tail = F))

ct_std2 <- difftime(Sys.time(), start_t,units = "secs") # stop the clock
```

Numerical Methods

When closed-form expressions for the quantile function, such as the `qexp` function, are unavailable, we could numerically evaluate the integrals and function inverses to produce a quantile function.

In the following code, we define a new exponential survivor function `f_surv` by first defining the hazard function, and then integrating the the hazard to produce the cumulative hazard. We can then compute the survival probabilities by exponentiating the negative cumulative hazards. The `f_surv(q, theta)` function numerically approximates the analytic exponential survivor function `pexp(q, rate=theta, lower.tail=FALSE)` in base `R`.

```
# exponential survivor function
f_surv <- function (q, theta) {
  f_haz <- hexp # exponential hazard function
  f_cum_haz <- function (q) { # cumulative hazard function
    integrate(f_haz, 0, q, rate = theta)$value
    }
  f_cum_haz <- Vectorize(f_cum_haz)
  cum_haz <- f_cum_haz(q)
  surv <- exp(-cum_haz) # survival probabilities
  return(surv)
}
```

We can invert the survivor function using the `qgeneric` function in `flexsurv` to produce the quantile function. We can then simulate survival times by evaluating the quantile function at a random uniform sample.

```
start_t <- Sys.time() # start the clock

# evaluate the survivor function at t_obs given theta
p_2 <- sapply(theta, function (x) f_surv(t_obs, x))

# sample random numbers on the interval [0, p_2]
r_num <- sapply(1:K, function (x) runif(n, 0, p_2[,x]))

# evaluate the quantile function given theta at the random uniform values
times_std3 <- sapply(1:K, function (x) {
  sapply(r_num[,x], function (y) qgeneric(f_surv, p = y, theta=theta[x]))
})

ct_std3 <- difftime(Sys.time(),start_t,units = "secs") # stop the clock
```

Interpolated Inverse Transform Sampling

We can also use inverse transform sampling to simulate survival times from the vectors of survival probabilities over discrete time units that we generated in the probabilistic analysis. Similar to the standard inverse transform sampling method, we first sample random uniform numbers. We then interpolate the survival probabilities using monotone cubic splines at the sampled numbers and record the interpolated cycle times

```
start_t <- Sys.time() # start the clock

# interpolate the model cycles at t_obs and record the survival probabilities
p_2 <- sapply(1:K, function (x) {
  spline(x=cycles, y=surv[,x], xout=t_obs, ties=max, method="hyman")$y
})

# sample random numbers on the interval [min(s), p_2]
r_num <- sapply(1:K, function (x) runif(n, min(surv[,x]) , p_2[,x]) )

# interpolate the survival probabilities at the random uniform numbers
# and record the corresponding cycle time
times_int <- sapply(1:K, function (x) {
  spline(x=surv[,x], y=cycles, xout=r_num[,x], ties=max, method="hyman")$y
})


ct_int <- difftime(Sys.time(),start_t,units = "secs") # stop the clock
```

Discrete Sampling

An alternative approach for simulating survival times from the vectors of survival probabilities over discrete time units is by sampling discrete 'bins' of cycle times with probability equal to the cumulative density of each bin. We can estimate the cumulative density of each bin by computing the successive differences between the survival probabilities at each discrete cycle time, which we then use as probabilities to sample the half-cycle times.

```
start_t <- Sys.time() # start the clock

# define the minimum and maximum half-cycle times
t_min <- round(mean(t_obs),0) + 0.5
t_max <- max(cycles-0.5)

# sample from the half-cycle times with probability equal to the
# successive differences between the survival probabilities at each cycle time
times_dis <- sapply(1:K, function (x) {
  sample(t_min:t_max, size = n,replace = TRUE,
         prob = abs(diff(surv[,x]))[ceiling(t_min):length(abs(diff(surv[,x])))])
})

ct_disc <- difftime(Sys.time(),start_t,units = "secs") # stop the clock
```

Results

Kernel Density Plot

The kernel density plot in Figure A1 shows that the distribution of the simulated survival times is very similar across the different methods.

```
df_times <- gather(as.data.frame(
  cbind(
    "Standard ITS (analytical)" = c(times_std2),
    "Standard ITS (numerical)" = c(times_std3),
    "Interpolated ITS" = c(times_int),
    "Discrete sampling" = c(times_dis)
  )
), factor_key = TRUE)

ggplot(df_times, aes(x=value, color = key)) +
  geom_density(alpha=0.4) +
  xlab("Simulated Survival Times") +
  ylab("Density") +
  labs(color = "Method")
```

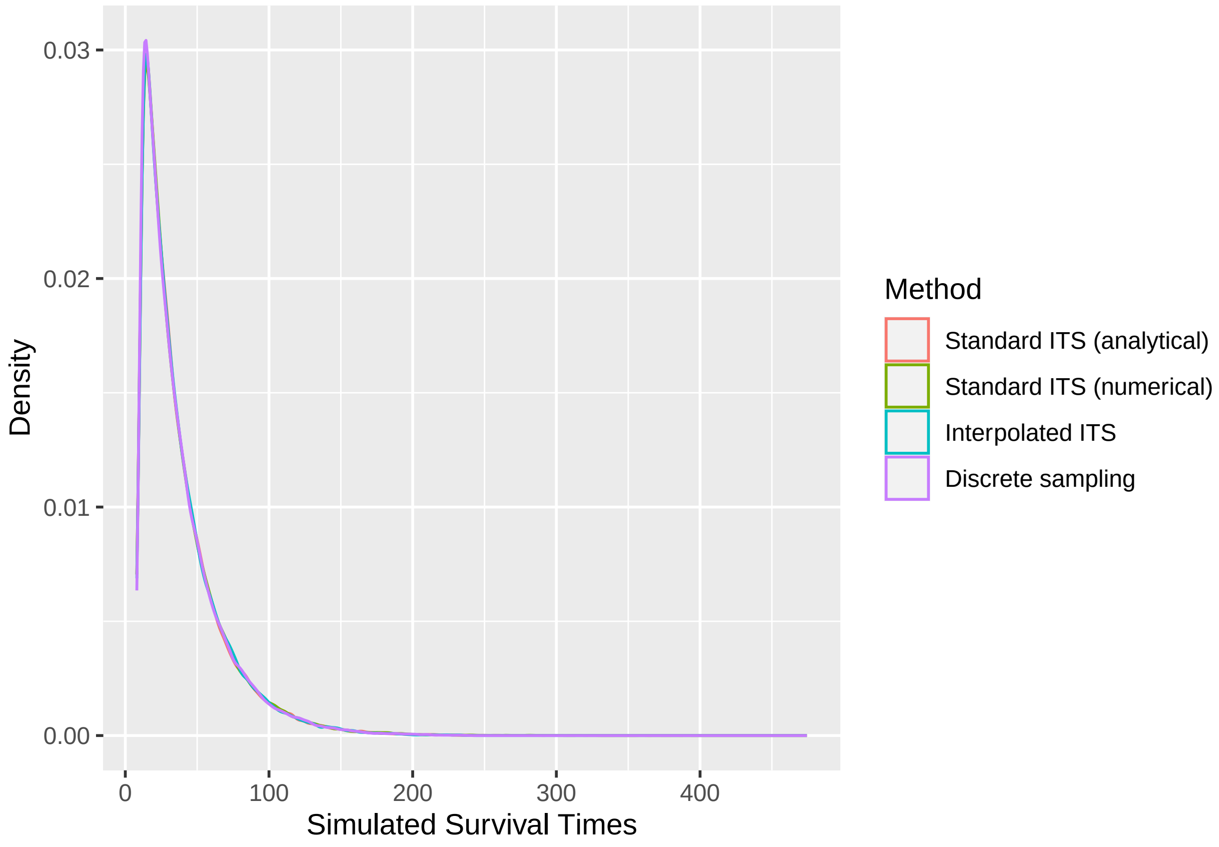

Figure A1: Density plot of the simulated survival times for the exponential example

Computation Time

The computation time for the different data-simulation procedures is given in Table A1. The standard inverse transform sampling scheme that uses numerical methods for evaluating the quantile function is several orders of magnitude slower than the other methods.

```
df_ct <- round(rbind(ct_std2, ct_std3, ct_int, ct_disc), 2)
rownames(df_ct) <- unique(df_times$key)
colnames(df_ct) <- "Computation time in seconds"
knitr::kable(df_ct,
             booktabs = TRUE,
             caption = "Computation times for the data-simulation procedures for the exponential example
             ) %>%
    add_footnote(" ",
                 threeparttable = T,
                 notation = "none")
```

Table A1: Computation times for the data-simulation procedures for the exponential example

	Computation time in seconds
Standard ITS (analytical)	0.02
Standard ITS (numerical)	271.26
Interpolated ITS	0.35
Discrete sampling	0.03

Appendix B - Synthetic case study dataset

We generated a synthetic dataset using evenly spaced OS and PFS times for each d given 4 Weibull distributions, using the $0.005^{th}, 0.015^{th}, ..., 0.985^{th}, 0.995^{th}$ quantiles from each distribution. This avoids generating implausible datasets where PFS exceeds OS or OS for standard care exceeds OS for the new treatment, which could occur by randomly sampling survival times. We enrolled all patients at time zero and right censored the survival times at 24 months. We did not apply any other type of censoring. The parameters of the Weibull distributions that we used to generate the synthetic case study data set are shown in Table B1, and the Kaplan-Meier plot of the synthetic case study data set is given in Figure B1.

Table B1: Weibull distribution parameters for the synthetic case study dataset

	New treatment	Standard care
Overall survival		
Weibull log-shape, α	0.30	0.35
Weibull log-scale, β	4.10	3.85
Progression-free survival		
Weibull log-shape, α	0.15	0.20
Weibull log-scale, β	3.60	3.30

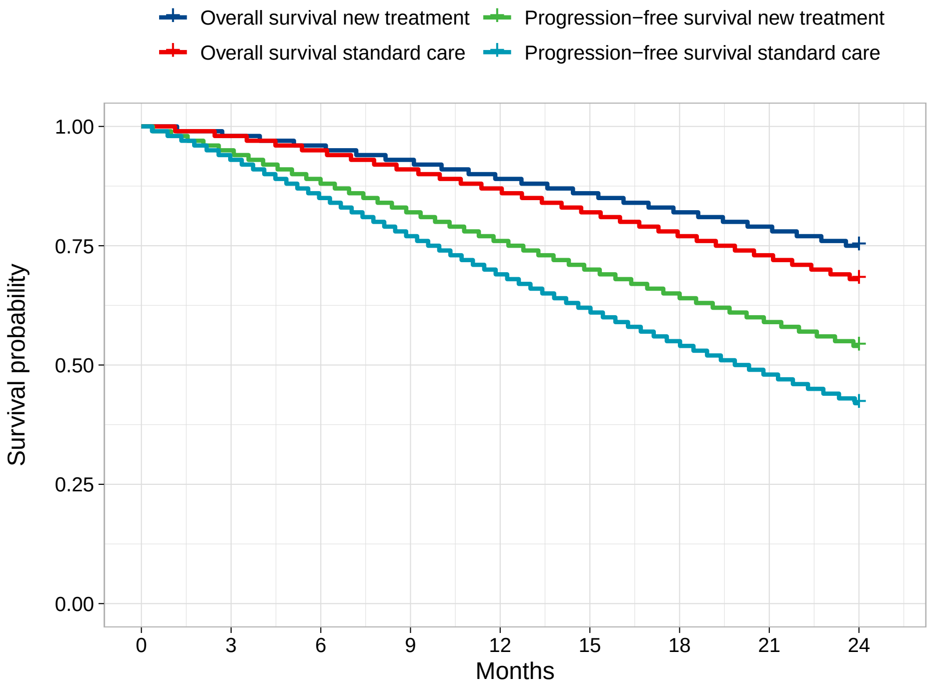

Number at risk

100	98	96	93	89	86	82	79	75
100	98	95	91	87	82	77	73	68
100	95	88	82	76	70	65	59	54
100	93	85	77	69	62	55	48	42

Figure B1: Kaplan-Meier plot for the synthetic case study dataset

Appendix C - Net benefit functions for the synthetic case study

The net benefit function for the new treatment is

$$\begin{aligned}\mathrm{NB}_1(\boldsymbol{\theta}) = \ & \frac{\lambda}{12} \sum_{c=0}^{C} e^{\left(-\left[\sqrt[12]{1+r}-1\right]\, t_c\right)} \left\{U_{pfs}\; S(t_c, \boldsymbol{\theta}_{pfs1}) + U_{pps}\left[S(t_c, \boldsymbol{\theta}_{os1}) - S(t_c, \boldsymbol{\theta}_{pfs1})\right]\right\} \\ & - \sum_{c=0}^{C} e^{\left(-\left[\sqrt[12]{1+r}-1\right]\, t_c\right)} \left\{C_{drug1}\; S(t_c, \boldsymbol{\theta}_{pfs1}) + C_{med1}\; S(t_c, \boldsymbol{\theta}_{os1})\right\},\end{aligned} \tag{1}$$

and the net benefit for standard care is

$$\begin{aligned}\mathrm{NB}_2(\boldsymbol{\theta}) = \ & \frac{\lambda}{12} \sum_{c=0}^{C} e^{\left(-\left[\sqrt[12]{1+r}-1\right]\, t_c\right)} \left\{U_{pfs}\; S(t_c, \boldsymbol{\theta}_{pfs2}) + U_{pps}\left[S(t_c, \boldsymbol{\theta}_{os2}) - S(t_c, \boldsymbol{\theta}_{pfs2})\right]\right\} \\ & - \sum_{c=0}^{C} e^{\left(-\left[\sqrt[12]{1+r}-1\right]\, t_c\right)} \left\{C_{med2}\; S(t_c, \boldsymbol{\theta}_{os2})\right\},\end{aligned} \tag{2}$$

where $S(t_c, \boldsymbol{\theta}_d)$ is the Weibull survivor function evaluated at cycle time t_c,

$$S(t_c, \boldsymbol{\theta}_d) = e^{-\left(t_c/e^{\beta_d}\right)^{e^{\alpha_d}}}. \tag{3}$$
